# Long-term follow-up of dogs with leishmaniosis treated with meglumine antimoniate plus allopurinol versus miltefosine plus allopurinol

**DOI:** 10.1186/s13071-015-0896-0

**Published:** 2015-05-28

**Authors:** Laura Manna, Raffaele Corso, Giorgio Galiero, Anna Cerrone, Paolo Muzj, Angelo Elio Gravino

**Affiliations:** Dipartimento di Medicina Veterinaria e Produzioni Animali, Università di Napoli Federico II, Via F. Delpino 1, 80137 Naples, Italy; Dipartimento di Sanità Pubblica, Università di Napoli Federico II, Via Pansini 5, 80131 Naples, Italy; Istituto Zooprofilattico Sperimentale del Mezzogiorno, Via Salute 2, 80055 Portici (Napoli), Italy

**Keywords:** Dog, Leishmaniosis, Meglumine antimoniate, Miltefosine, RT-QPCR

## Abstract

**Background:**

Visceral leishmaniosis is a potentially life-threatening illness caused by a protozoan parasite of the genus *Leishmania*. It is found mainly in areas where both the parasite and its vector are endemic and is one of the most challenging infectious diseases in the world to control. HIV infected patients are vulnerable to *Leishmania* infections, and the main reservoir hosts of *Leishmania infantum* parasites are domestic dogs. Here, we evaluated the long-term efficacy of treatment with meglumine antimoniate plus allopurinol (G1) compared to miltefosine plus allopurinol (G2) in dogs naturally infected *L. infantum*.

**Methods:**

Eighteen dogs with leishmaniosis were divided into the following two groups: G1 (n = 9) was treated subcutaneously with meglumine antimoniate (100 mg/kg/day/30 days) plus allopurinol (10 mg/kg/per day/30 days), while G2 (n = 9) was treated orally with miltefosine (2 mg/Kg/day/30 days) plus allopurinol (10 mg/kg/day/30 days). Thereafter, the same dose of allopurinol was administered to both groups for 6 years. *Leishmania* DNA in lymph node aspirates from the G1 and G2 dogs was quantified by real-time quantitative PCR at baseline and every 3 months for 24 months, and then at 28, 36, 48, 60 and 72 months. At each assessment, the dogs were examined for signs of disease, and their clinical scores were recorded.

**Results:**

Both combination therapies produced significant clinical improvements in the dogs, with a significant reduction in the parasitic load in the lymph nodes of the dogs from both groups after 3 months of treatment. Clinical relapses were observed in four dogs from G2 (miltefosine/allopurinol), and just one dog from G1 (meglumine antimoniate/allopurinol). All dogs that relapsed had increased clinical scores, and increased anti-*Leishmania* antibody titers and parasitic loads in their lymph nodes.

**Conclusions:**

Long-term, the clinical and laboratory findings of the G1 dogs were more stable than those of the G2 dogs, thus indicating that meglumine antimoniate had better clinical efficacy than miltefosine. The results suggest that treatment with allopurinol as a maintenance therapy is crucial for stabilizing the care of canine leishmaniosis.

**Electronic supplementary material:**

The online version of this article (doi:10.1186/s13071-015-0896-0) contains supplementary material, which is available to authorized users.

## Background

Leishmaniosis is a protozoan infection of dogs, and *Leishmania infantum* (syn. *L. chagasi*) is the most important etiological agent [1].

Several drugs used to treat the disease are able to temporarily improve the clinical signs or clinically cure dogs, but none of these treatments reliably eliminates the infection. The most commonly used treatments for CanL are a combination of meglumine antimoniate plus allopurinol, or miltefosine plus allopurinol. Some studies have shown that these drugs, alone or in combination, can clinically cure most dogs of the disease, but they do not lead to complete elimination of the parasite [2–4].

Miltefosine, a phospholipid (hexadecyl-phosphocholine) originally developed as an oral antineoplastic agent, has been registered in India for the treatment of human visceral leishmaniosis since March 2002 [5]. It is the first and still remains the only oral drug that can be used to treat leishmaniosis [6]. The drug was chosen for the elimination of leishmaniosis in India, Nepal and Bangladesh because of its ease of application in parasite control programs [7]. However, recent studies have shown that its efficacy appears to have declined because its relapse rate has doubled [8]. Additionally, our previous study showed that treatment with miltefosine alone reduced *Leishmania* replication but the parasite was not completely removed from the lymph nodes. For this reason, the action of miltefosine for the treatment of canine leishmaniosis appears to be ineffective [9].

In most parts of the world, the most widely used treatment for human and canine leishmaniosis is meglumine antimoniate [10]. Meglumine antimoniate, which has a parasiticidal activity and also potentiates the phagocytic capacity of macrophages [11], causes a marked decrease in the parasite load in dogs during the first four weeks of treatment [3–12]. In contrast, allopurinol has a parasitostatic activity and long-term administration keeps the parasite load low thereby preventing relapses [13, 14]. Previous studies have shown that in combination, miltefosine and allopurinol have similar efficacy as the ‘’gold standard” treatment with meglumine antimoniate and allopurinol; however, the follow-up period for these studies was less than seven months [15].

In the present study, to evaluate the efficacy of the two treatments, meglumine antimoniate plus allopurinol versus miltefosine plus allopurinol, we monitored 18 dogs with leishmaniosis for 6 years to determine if the disease could be eradicated in them.

## Methods

### Ethical statement

Dogs with leishmaniosis were treated and monitored according to the guidelines for the control of canine leishmaniosis issued by the Regione Campania, which provides compulsory medical treatment for dogs and the periodic monitoring of dogs in this part of Southern Italy. All dogs in this study were infected with leishmaniosis and were, therefore, treated according to the regional legislation and the involvement of the Animal Welfare Committee. Written consent from all dog owners was obtained to allow us to perform clinical evaluations of the dogs, as well as collecting blood and lymph node aspirate samples at the time of diagnosis and during the post-therapy follow-up. All the procedures were performed in the presence of the dog owners and good veterinary practice was used to avoid suffering in the dogs. Also, in this retrospective study no dogs were sacrificed.

### Inclusion criteria

The trial was performed using 18 dogs with leishmaniosis hospitalized at the Department of Veterinary Medicine and Animal Productions of the University of Naples Federico II (Naples, Italy). All the dogs were enrolled between May 2001 and January 2013 and were followed-up for at least 6 years. The diagnosis of leishmaniosis in the dogs was based on the clinical manifestations, a positive immunofluorescence test (IFAT) the presence of anti-*Leishmania* serum antibodies, and a positive real-time quantitative PCR assay (RTQ-PCR) to determine the presence of the parasite and estimate its DNA load.

### Exclusion criteria

Dogs with renal failure or with a concomitant disease that might interfere with evaluation of treatment responses were excluded. Additionally, ehrlichiosis was ruled out by a specific *Ehrlichia canis* IFAT, and by clinical examination and hematological tests (thrombocytopenia and leukocytosis). Dogs previously treated with leishmanicidal or leishmaniostatic drugs before inclusion were also excluded.

### Visit schedule and sample collection

Before therapy, all the dogs enrolled in the study showed clinical signs and clinic-pathological abnormalities characteristic of CanL at the time of diagnosis. Post-therapy assessments were performed on day 30 and thereafter at 3, 6, 9, 12, 15, 18, 21, 24, 28, 36, 48, 60, and 72 months.

Before therapy and during the post-therapy follow-up, and with the consent of the dog owners, blood and lymph node aspirate samples were collected from the dogs. Clinical evaluation of the dogs and their hemato-biochemical profiles (including blood cell counts; aspartate transaminase, AST; alanine aminotransferase, ALT; total serum proteins, creatinine, urea, albumin/globulin ratio, urine analysis, and IFAT) were recorded. Dogs were scored based on the presence and severity of signs attributable to CanL (i.e., weight loss, dermatitis, skin ulcers, alopecia, ocular lesions, generalized lymphadenomegaly, splenomegaly, epistaxis, hemorrhagic diarrhea, fever, apathy, anorexia, orchitis, lameness, hematuria, bone lesions, and liver involvement), the laboratory parameters, and the sum of the values were recorded to give a clinical score as described previously [9]. In addition, imaging techniques, such as radiography and/or echography, were appropriately performed in all cases in which other concomitant causes were suspected. The dogs were also classified following the clinical guide lines proposed by Solano Gallego *et al.* [16].

### Treatment

With the agreement of their owners, the study dogs were allocated to two treatment groups. The orally administered drug treatment was preferable where there was a risk of a dog bite, while the parenteral drug treatment was preferred in dogs which were not controlled during the early stages of administration to avoid the possibility that nausea caused expulsion of the drug via regurgitation or vomiting.

The two treatment groups were as follows: Group 1 (G1) contained nine dogs with leishmaniosis treated with meglumine antimoniate (100 mg/kg/per day/30 days, subcutaneous) plus allopurinol (10 mg/kg/per day/sine die; per os, PO); Group 2 (G2) contained nine dogs with leishmaniosis treated with miltefosine (2 mg/Kg/per day/30 days, PO) plus allopurinol (10 mg/kg/per day/sine die, PO). After 30 days of combined therapy, allopurinol was continued at the same dose until the end of the study period (6 years). Thus, the dogs received allopurinol for the entire 72-month study period. G1 and G2 each contained one dog where itching was observed as a side-effect. In both cases we decided to discontinue the treatment for 1 month before recommencing allopurinol administration. Relapsing dogs were re-treated with meglumine antimoniate/miltefosine in combination with allopurinol for another 30 days.

### Efficacy of treatment: clinical outcome and laboratory analyses

With the consent of the dog owners, the efficacy of each therapy was evaluated at each time-point based on the clinical and laboratory responses to treatment of each dog.

### Efficacy of treatment: parasite burden

At each time point, the *Leishmania* DNA load in the lymph node aspirates was determined by RTQ-PCR analysis of the parasite DNA using a method described previously [3].

### Statistical methods

All data are reported as the average, standard deviation (SD), and standard error (SE). Statistical analyses of the data were performed using a Student's *t*-test and an analysis of variance (ANOVA); p values less than 0.05 were considered statistically significant.

## Results

The clinical scores and serological data for the two populations in each treatment group were recorded over time. We also recorded changes in the parasitic load in the lymph nodes before and after therapy.

### Clinical outcomes and laboratory analyses

Clinical examination of both study groups was carried out before therapy and at all times during the follow-up as indicated in the Material and Methods section. The clinical scores, IFAT results, and the *Leishmania* DNA load recorded in this study are shown in Tables 1, 2, and 3, respectively. The clinical score for each dog (obtained following the guide lines proposed by Solano Gallego [16] is shown in Additional file 1: Table S1, in which the basal state clinical evaluation was compared with that obtained by the Poot method [17], as was performed herein. The hemato-biochemical alterations observed most frequently included moderate anemia, thrombocytopenia, leukopenia, increased β-γ globulins, and hepatic enzymes.

**Table 1:** Clinical score changes in G1 and G2 treated dogs during follow-up post-therapy. Data are reported as mean and (SD)

Time course	G1	G2	p*
months	n = 9	n = 9	
Basal	6.2	6.0	0.3894
	(1.6)	(1.7)	
1	1.7	3.7	0.0051
	(1.7)	(1.2)	
3	1.3	1.7	0.1922
	(1.0)	(0.5)	
6	0.6	2.3	0.0246
	(0.7)	(2.4)	
9	0.0	1.0	0.0380
	(0.0)	(1.6)	
12	0.9	0.4	0.3180
	(2.7)	(0.7)	
15	0.2	0.1	0.3304
	(0.7)	(0.3)	
18	0.1	0.3	0.1418
	(0.3)	(0.5)	
21	0.1	0.3	0.1418
	(0.3)	(0.5)	
24	0.2	0.3	0.3119
	(0.4)	(0.5)	
28	0.3	0.9	0.0862
	(0.7)	(0.9)	
36	0.0	0.4	0.0111
	(0.0)	(0.5)	
48	0.0	0.8	0.0243
	(0.0)	(1.1)	
60	0.0	0.4	0.0750
	(0.0)	(0.9)	
72	0.0	0.4	0.0426
	(0.0)	(0.7)	

*Student’s *t*-test probability significance between groups

**Table 2:** IFAT score changes in G1 and G2 treated dogs during follow-up post-therapy. Data are reported as mean and (SD)

Time course	G1	G2	p*
months	n = 9	n = 9	
Basal	2.8	3.3	0.1093
	(1.1)	(0.7)	
1	1.3	3.3	0.0001
	(0.9)	(0.7)	
3	2.6	2.3	0.2818
	(0.5)	(1.0)	
6	1.6	3.2	0.0004
	(0.7)	(1.0)	
9	0.9	1.2	0.1408
	(0.3)	(0.8)	
12	1.1	1.0	0.4050
	(1.2)	(0.7)	
15	1.0	1.2	0.2177
	(0.5)	(0.7)	
18	1.0	0.9	0.2934
	(0.0)	(0.6)	
21	0.9	1.0	0.3377
	(0.6)	(0.5)	
24	0.9	1.3	0.0537
	(0.6)	(0.5)	
28	1.0	1.6	0.1550
	(0.7)	(1.4)	
36	1.0	1.4	0.1277
	(0.9)	(0.7)	
48	1.0	1.6	0.0982
	(0.5)	(1.1)	
60	1.0	1.2	0.1661
	(0.0)	(0.7)	
72	0.3	0.8	0.0646
	(0.5)	(0.7)	

*Student’s *t*-test probability significance between groups

**Table 3:** DNA load changes in G1 and G2 treated dogs during follow-up post-therapy. Data are reported as mean and (SD)

Time course	G1	G2	p*
months	n = 9	n = 9	
Basal	4952	5222	0.4385
	(3341)	(3935)	
1	386	1723	0.0686
	(417)	(2528)	
3	94	149	0.1356
	(84)	(120)	
6	91	944	0.1255
	(79)	(2148)	
9	76	184	0.1114
	(19)	(253)	
12	795	88	0.1687
	(2140)	(153)	
15	71	56	0.2475
	(17)	(63)	
18	43	32	0.2173
	(16)	(36)	
21	36	37	0.4520
	(19)	(36)	
24	33	778	0.1529
	(18)	(2112)	
28	16	1155	0.1448
	(7)	(3119)	
36	14	256	0.1464
	(5)	(667)	
48	4	756	0.1622
	(3)	(2220)	
60	7	114	0.1444
	(4)	(292)	
72	16	98	0.1616
	(27)	(243)	

*Student’s *t*-test probability significance between groups

Table 1 shows the pre-therapy scores for the clinical basal state of both groups of dogs based on the presence and severity of the clinical signs and laboratory parameters. The scores for both groups were comparable and not statistically different (6.2 vs 6.0 as averages), while the inter-individual variability (standard deviation) of the groups was 1.6 for G1 and 1.7 for G2. The results of the ANOVA (with Bonferroni post-hoc test) among all scores showed that the basal state was significantly higher than all of the other time points (from 1 to 72 months) in both treatment groups. After thirty days of therapy with meglumine antimoniate or miltefosine, an improvement in the clinical condition of both groups was observed, even though the scores were significantly reduced in G1 dogs where the score decreased an average of 4.5 points (from 6.2 to 1.7) compared to the G2 dogs where the score reduced by 2.3 points (from 6.0 to 3.7). Conversely, the G2 clinical score by ANOVA one month after therapy was significantly higher than all the other time points (from 3 to 72 months). In this analysis, just one comparison was not statistically significant (1 month *vs* 6 months) and this was related to a relapse in two of the dogs. In G1 a dog relapsed after 12 months while the other subjects were asymptomatic. In contrast, there were no observable relapses in the G2 dogs after 12 months, and there was an improvement in their symptoms compared to the previous months; however, only six out of nine dogs were clinically cured. From month 15, all the G1 dogs were clinically cured. Contrastingly, two G2 dogs relapsed at 28 and 48 months. Hence, a second 30-day cycle of the drug, antimoniate or miltefosine, was administered to the relapsed subjects in the G1 (n = 1) and G2 (n = 4) groups, respectively.

**Fig. 1:**
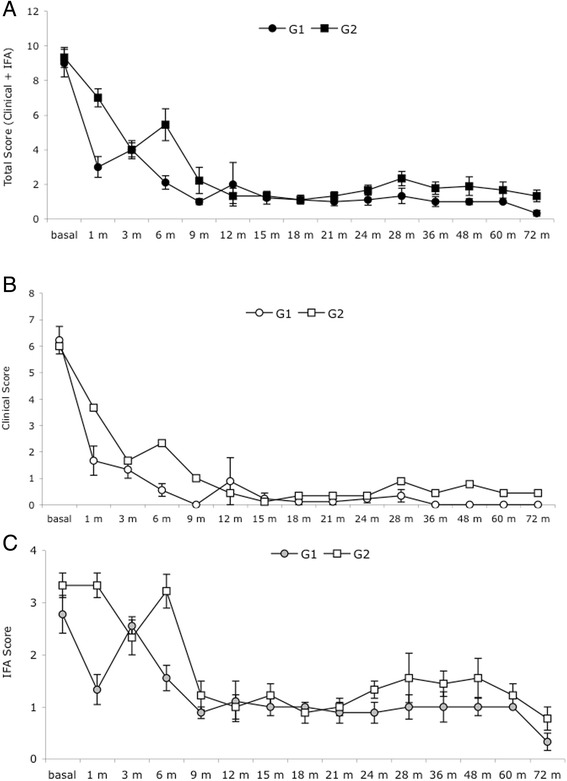
Clinical and laboratory scores for G1 and G2 dogs during the post-therapy follow-up period. The scores were recorded at baseline, then every three months for 24 months, and then at 28, 36, 48, 60 and 72 months. The data for (**a**) total score (clinical + IFAT score), (**b**) clinical score, and (**c**) IFAT score. Scores are reported as the average and standard deviation

The IFAT scores in Table 2 show that in both groups the anti-*Leishmania* antibody titers in the blood decreased progressively from 2.8 (basal) to 0.3 (72 months) in G1 and from 3.3 to 0.8 in G2. However, the anti-*Leishmania* antibody levels in G1 were consistently lower than G2 as early as 6 months during the follow-up period. In G2, however, we observed a rise in the antibody titers in conjunction with the clinical relapses. A comparison of the clinical and IFAT scores is shown in Fig. 1. The total score obtained from the sum of the clinical and IFAT values is also shown in Fig. 1, panel a.

The IFAT score for the basal state was significantly higher than all the other time points (from 1 to 72 months) in G1 and just one comparison was not significant (basal *vs* 3 months). The G2 IFAT score for the basal state was significantly higher than all the other time points (from 9 to 72 months); however, the basal score was not significantly different for the 1, 3 and 6 months post-therapy values (by ANOVA).

At 30 days post-therapy with meglumine antimoniate or miltefosine, the IFAT value decreased by 1.5 points (from 2.8 to 1.3) in G1, whereas the G2 score was unchanged (from 3.3 vs 3.3). The G2 IFAT value after 1 month of therapy was significantly higher (by ANOVA) than all the other time points (from 9 to 72 months), and just two comparisons were not significant (1 month *vs* 3 and 6 months).

In G1, the IFAT after 3 months was significantly higher than all the other time points (from 9 to 72 months) and just one comparison was not significant (3 *vs* 6 months). The G2 IFAT after 3 months was not significantly higher than all the other time points (from 6 to 72 months) and just two comparisons were significant (3 vs 18 and 72 months). In G1, the IFAT score at 6 months compared to all the other scores showed just one value that was significantly higher (6 *vs* 72 months). In contrast, the IFAT scores of the G2 dogs at the 6-month evaluation were significantly higher than all the other time points (from 9 to 72 months).

**Fig. 2:**
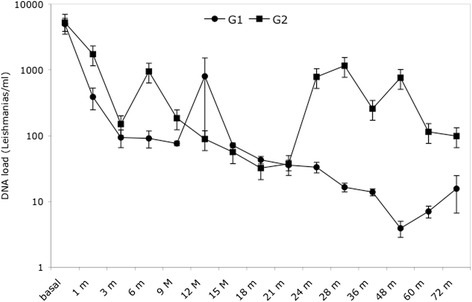
*Leishmania* DNA load in G1 and G2 dogs. The parasite load in the lymph node during the post-therapy follow-up was measured by RTQ-PCR. Data are reported as the logarithms of the average values and their standard deviations

The quantitative measurements of the DNA in the lymph nodes did not differ significantly in either group at baseline, and on average, the values of *Leishmania* DNA per ml of aspirate for the G1 and G2 dogs were 4952 (range 981–9100) and 5222 (range 159–10,000), respectively. Table 3 shows that the *Leishmania* load before therapy in both groups of dogs was not statistically different (4952 *vs* 5222, average values). Inter-individual variability between the groups (standard deviation) was 3341 in G1 and 3935 in G2 (Table 3). The score for the basal state compared to all the other time points (from 1 to 72 months) was significantly higher in both groups of dogs (by ANOVA). Figure 2 shows that in both groups the *Leishmania* load after therapy decreased strongly, particularly after 30 days of therapy. Additionally, the parasite load decreased on average by 4565 parasites (from 4952 to 386) in G1 compared to G2 where the load was reduced by 3500 parasites (from 5222 to 1723). The ANOVA also showed that the *Leishmania* load after 1 month of therapy in both groups of dogs did not differ significantly for all the time points (from 3 to 72 months). The decrease in parasite load for G1 was already statistically significant after just one month of treatment, with a linear decrease in the subsequent nine months reaching an average value of 76 *Leishmania* per ml of aspirate (range 42–98). In contrast, the G2 dogs showed higher variability within their group (compared with G1), and at 9 months had an average value of 184 *Leishmania* per ml of aspirate (range 35–698).

In both groups at 3 months after commencing treatment, the parasite load did not differ statistically from each other, and the decreased parasite load in G1 and G2 was about 50 and 35 times lower than the initial values, respectively. At 12 months an increase in the *Leishmania* DNA load was apparent in G1, the average value of which was higher than in G2. In particular, there was higher variability within G1; this was caused by a relapse in one of the dogs that had an increased DNA load (6501 L/ml of aspirate). From months 15 to 21, the *Leishmania* DNA load for both groups was stable and much lower than that of the basal state.

From month 24, the *Leishmania* DNA load in the G1 and G2 dogs differed from each other. In fact, the DNA load in G2 was consistently higher than that in G1, but not statistically different. Additionally, G2 showed more variability in the DNA load compared with G1. However, in all cases the DNA loads for G1 dogs from month 24 onwards were below 33 parasites per ml of aspirate. In particular, for three of the G1 dogs during the 48 to 72 month period, the DNA load was below three parasites per ml of aspirate, and only one dog had an undetectable *Leishmania* load at 72 months post-treatment.

## Discussion

This longitudinal study involved constant and systematic monitoring of 18 dogs with leishmaniosis over 6 years in order to evaluate the efficacy of two different treatments, meglumine antimoniate/allopurinol versus miltefosine/allopurinol. It is the first comparative study of two different drug combinations performed by evaluation of the clinical parameters, the hemato-biochemical profiles (urea, serum total protein, creatinine, globulins and albumin, AST, ALT), the serological (IFAT), and molecular (RTQ-PCR) data.

Clinical improvement occurred rapidly in the G1 dogs (30 days after meglumine antimoniate/allopurinol therapy) and less rapidly in the G2 dogs (3 months after miltefosine/allopurinol therapy). However, in agreement with previous reports [14, 18, 19], almost all the dogs in both groups were free of clinical signs at 90 days after drug treatment.

The incidence of clinical recurrence was higher in the G2 dogs than in the G1 dogs from 12 months after starting therapy. Indeed, in G1, eight of nine dogs were clinically cured, and by month 12 only one dog had relapsed. From month 15, all G1 dogs were clinically cured. However, in G2, two dogs relapsed at 6 months and two dogs relapsed at 28 and 48 months. RTQ-PCR analysis of parasite DNA in the lymph nodes of the dogs with leishmaniosis treated with a combination of meglumine antimoniate and allopurinol showed there was a significant decrease in the parasite load of the G1 dogs after only one month of therapy, with a linear decrease observed in the subsequent nine months. In particular, in three dogs from this group the DNA load in their lymph nodal aspirates was below 3 *Leishmania* parasites per ml of aspirate from 48 months to the end of the observation period (6 years). Furthermore, the DNA load of the parasite in one dog was undetectable at 72 months. These values are considered negligible when compared to the initial values, and are in agreement with those reported previously in infected dogs from areas where *Leishmania* is endemic; dogs from endemic regions react to the parasite by immune response activation, and thereby remain clinically healthy or asymptomatic for long periods [20]. In contrast, G2 dogs that received miltefosine plus allopurinol showed greater variability within the group, and although the parasite load in both groups was similar at 3 months, from month 24 the *Leishmania* DNA load showed a different trend between the two groups of dogs with the number of parasites in G2 being higher than that of G1 (not statistically significant).

In this study, we found a positive correlation between the clinical score and the antibody titer (IFAT score) in both groups of dogs (G1 and G2), with correlation factors (r) of 0.802 and 0.877, respectively. We also found a positive correlation between the total score (clinical score + IFAT score) and parasite load in both groups (G1 and G2) with correlation factors (r) of 0.917 and 0.861, respectively. Also, the IFAT score versus the parasite load showed a positive correlation for both G1 and G2 dogs, with correlation factors (r) of 0.679 and 0.705, respectively. Additionally, as has been suggested by Abranches *et al.* (1991) [21], there was a positive correlation between the clinical score and parasite load in G1 and G2 dogs alike, with correlation factors (r) of 0.964 and 0.915, respectively. All the correlations were statistically significant (p < 0.05). The supplementary data (Additional file 1: Figure S1 and S2) shows the correlations identified for the clinical and laboratory parameters that were investigated in the G1 and G2 dogs. These results are in agreement with some studies that have indicated the existence of a close correlation between the clinical response and IFAT score reduction after therapy [14, 22]. The results differ from other studies that have shown that IFAT titers can be high in clinically negative dogs [23]. Here, in both G1 and G2 groups, excluding the findings for G1 after 72 months, all the dogs had antibodies against *Leishmania* parasites, and the IFAT scores in both groups correlated positively with their clinical scores and parasite loads. Therefore, serology does not seem to be a reliable way to monitor treatment efficacy in the short-term [23]. There was no correlation between the serological titers and the severity of the clinical signs. It is concluded that the ELISA is a sensitive method for the diagnosis of canine leishmaniosis but is not satisfactory for monitoring the clinical development of the disease [23]. High antibody levels are associated with high parasitism and disease [24]. However, the presence of low antibody levels is not necessarily indicative of the disease and further work-up is necessary to confirm or exclude clinical leishmaniosis by other diagnostic methods [4].

Notably, during the 72-month follow-up period, in all the relapsed dogs (1 dog in G1 and 3 dogs in G2) a rise in antibody titers was observed in conjunction with the clinical relapses. According to Reis *et al.* [24], our data confirm that IFAT is suitable for identifying *Leishmania*-infected dogs, irrespective of their clinical status. However, when the clinical signs are evident, the antibody levels increase significantly [21]. Based on these results, and on the direct correlation between parasite load and disease severity observed here, we suggest that the RTQ-PCR method is suitable for monitoring changes in the parasitic load during post-therapy follow-up, and may be an effective way to confirm accurately the presence of parasites in dog tissues. This study also begins to address the question of appropriate allopurinol dose during long-term follow-up.

Contrary to the study by Torres *et al.* [14], here, allopurinol was administered for the entire observation period at a dose of 10 mg/kg/per day. No symptomatic dogs had urolithiasis, as determined by use of ecographic scanning of the uropoietic system and by urine analysis (for blood and microscopic sediment). Based on these findings, we suggest that a single daily administration of allopurinol at a dose of 10 mg/kg/day may promote greater tolerance of this drug. Furthermore, increasing the administration period by up to 6 years should not cause significant side effects, as was noted in the present study. In fact, only one dog in G1 and one in G2 had itching as a side effect due to a long allopurinol administration period, suggesting possible hypersensitivity of the two dogs as also observed in humans [25–27]. In both cases the treatment was discontinued for one month, after the allopurinol administration was started again.

## Conclusions

After the 6-year follow-up in each treatment group (G1 and G2), the average clinical scores and *Leishmania* loads were significantly lower than before starting therapy. The effects of meglumine antimoniate plus allopurinol seem better than miltefosine plus allopurinol for treating leishmaniosis in dogs, because during the follow-up we observed a decrease in the incidence of disease recurrence. Indeed, one of nine dogs had a recurrence in the meglumine antimoniate plus allopurinol group (G1), compared to four of nine dogs that received miltefosine plus allopurinol (G2). Because a relatively small number of dogs were used in this study, it would be worth repeating it with a larger number of dogs.

## Additional file

Additional file 1:
**Additional data.**
