# Supplementary material for: Long-term follow-up of dogs with leishmaniosis treated with meglumine antimoniate plus allopurinol versus miltefosine plus allopurinol
Source: Parasit Vectors. 2015 May 28;8:289. doi: 10.1186/s13071-015-0896-0 (PMC4458061; doi:10.1186/s13071-015-0896-0)
Supplement: Additional file 1: — Additional data. [file 13071_2015_896_MOESM1_ESM.doc]

ADDITIONAL DATA

**Long-term follow-up of leishmaniotic dogs treated with meglumine antimoniate plus allopurinol versus miltefosine plus allopurinol**

Laura Manna^1*^, Raffaele Corso^2^, Giorgio Galiero^3^, Anna Cerrone^3^, Paolo Muzj^1^, Angelo Elio Gravino^1^

^1^Dipartimento di Medicina Veterinaria e Produzioni Animali, Università di Napoli Federico II, Via F. Delpino 1, 80137 Napoli, Italy

^2^Dipartimento di Sanità Pubblica, Università di Napoli Federico II, Via Pansini 5, 80131 Napoli, Italy

^3^Istituto Zooprofilattico Sperimentale del Mezzogiorno, Via Salute 2, 80055 Portici (Napoli), Italy

Correspondence: laumanna@unina.it

Laura Manna, M.V.D.

Department of Veterinary Medicine and Animal Productions

University of Naples Federico II

Via F. Delpino 1, 80137 Naples, Italy.

Tel: +39 081 2536006; Fax: +39 081 2536008

| Table S1: Clinical evaluation of studied dogs at basal state according to Poot (ref. 18) and Solano-Gallego (ref. 17). | | | | | | | | | | | |
| --- | --- | --- | --- | --- | --- | --- | --- | --- | --- | --- | --- |
| **Dogs** | **1** | **2** | **3** | **4** | **5** | **6** | **7** | **8** | **9** |  |  |
| **Signs of Group 1 (ref.18)** |  |  |  |  |  |  |  |  |  |  |  |
| lymphadenopaty | 2 | 1 | 1 | 2 | 1 | 1 | 1 | 2 | 1 |  |  |
| anorexia | 1 | 1 | 1 | 1 | 0 | 1 | 0 | 1 | 0 |  |  |
| apathy | 1 | 1 | 1 | 1 | 0 | 1 | 0 | 1 | 1 |  |  |
| skin envolvment | 2 | 0 | 0 | 0 | 0 | 0 | 0 | 0 | 1 |  |  |
| weight loss | 1 | 1 | 1 | 1 | 0 | 0 | 1 | 1 | 1 |  |  |
| haemorragic diarrhea | 0 | 0 | 0 | 1 | 0 | 0 | 0 | 0 | 0 |  |  |
| hypertermia | 0 | 0 | 0 | 0 | 0 | 0 | 0 | 0 | 0 |  |  |
| ocular lesions | 0 | 0 | 0 | 0 | 0 | 0 | 0 | 0 | 0 |  |  |
| haematuria | 0 | 0 | 1 | 0 | 0 | 0 | 0 | 0 | 0 |  |  |
| orchitis | 0 | 0 | 0 | 0 | 0 | 0 | 1 | 0 | 0 |  |  |
| lamness | 0 | 0 | 0 | 0 | 1 | 1 | 0 | 0 | 0 |  |  |
| iperprotinemia | 1 | 1 | 1 | 1 | 1 | 1 | 1 | 1 | 1 |  |  |
| epistaxis | 0 | 0 | 0 | 0 | 0 | 0 | 0 | 0 | 0 |  |  |
| proteinuria | 0 | 0 | 0 | 0 | 0 | 0 | 1 | 1 | 1 |  |  |
| prostatitis | 0 | 0 | 0 | 0 | 0 | 0 | 0 | 0 | 0 |  |  |
| trombocitopenia | 0 | 0 | 0 | 1 | 1 | 0 | 0 | 0 | 0 |  |  |
| lamness | 1 | 1 | 0 | 0 | 0 | 0 | 0 | 0 | 0 |  |  |
| several bone lesion | 0 | 0 | 0 | 0 | 0 | 0 | 0 | 0 | 0 |  |  |
| Hepatic lesion | 0 | 0 | 0 | 0 | 0 | 0 | 0 | 0 | 0 |  |  |
| Total score (ref. 18) | 9 | 6 | 6 | 8 | 4 | 5 | 5 | 7 | 6 |  |  |
|  |  |  |  |  |  |  |  |  |  |  |  |
| Staging (ref. 17) | 3 | 3 | 2 | 2 | 3 | 3 | 3 | 2 | 2 |  |  |
|  |  |  |  |  |  |  |  |  |  |  |  |
| **Dogs** | **1** | **2** | **3** | **4** | **5** | **6** | **7** | **8** | **9** |  |  |
| **Signs of Group 2 (ref. 18)** |  |  |  |  |  |  |  |  |  |  |  |
| lymphadenopaty | 2 | 1 | 1 | 1 | 1 | 1 | 1 | 1 | 1 |  |  |
| anorexia | 0 | 1 | 1 | 0 | 0 | 0 | 0 | 0 | 1 |  |  |
| apathy | 1 | 1 | 1 | 1 | 1 | 0 | 1 | 0 | 0 |  |  |
| skin envolvment | 1 | 0 | 1 | 1 | 0 | 0 | 1 | 0 | 1 |  |  |
| weight loss | 1 | 0 | 1 | 0 | 0 | 0 | 1 | 0 | 1 |  |  |
| haemorragic diarrhea | 0 | 0 | 0 | 0 | 0 | 0 | 0 | 1 | 0 |  |  |
| hypertermia | 0 | 0 | 0 | 1 | 0 | 0 | 1 | 0 | 0 |  |  |
| ocular lesions | 1 | 0 | 1 | 0 | 0 | 0 | 0 | 0 | 0 |  |  |
| haematuria | 0 | 1 | 0 | 0 | 0 | 0 | 0 | 0 | 0 |  |  |
| orchitis | 0 | 0 | 0 | 0 | 0 | 0 | 0 | 0 | 0 |  |  |
| lamness | 0 | 0 | 0 | 0 | 0 | 1 | 0 | 0 | 0 |  |  |
| iperprotinemia | 1 | 1 | 1 | 1 | 1 | 1 | 1 | 1 | 1 |  |  |
| epistaxis | 0 | 0 | 0 | 1 | 0 | 0 | 0 | 0 | 0 |  |  |
| proteinuria | 0 | 1 | 0 | 0 | 0 | 0 | 0 | 0 | 0 |  |  |
| prostatitis | 0 | 1 | 0 | 0 | 0 | 0 | 0 | 0 | 0 |  |  |
| trombocitopenia | 0 | 0 | 0 | 0 | 0 | 0 | 1 | 1 | 1 |  |  |
| lamness | 0 | 0 | 0 | 0 | 0 | 0 | 0 | 0 | 0 |  |  |
| several bone lesion | 0 | 0 | 0 | 0 | 1 | 0 | 0 | 0 | 0 |  |  |
| Hepatic lesion | 0 | 1 | 0 | 0 | 1 | 0 | 1 | 0 | 0 |  |  |
| Total score (ref. 18) | 7 | 8 | 7 | 6 | 5 | 3 | 8 | 4 | 6 |  |  |
|  |  |  |  |  |  |  |  |  |  |  |  |
| Staging (ref. 17) | 3 | 3 | 3 | 2 | 3 | 3 | 2 | 2 | 2 |  |  |


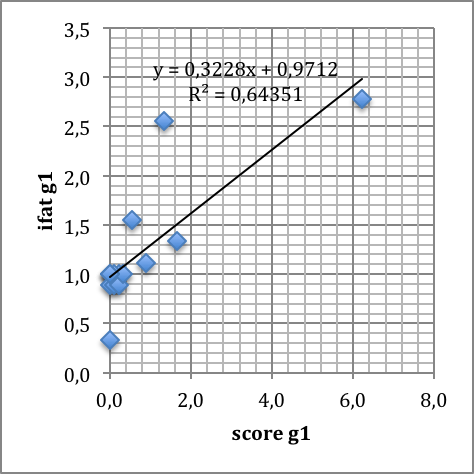

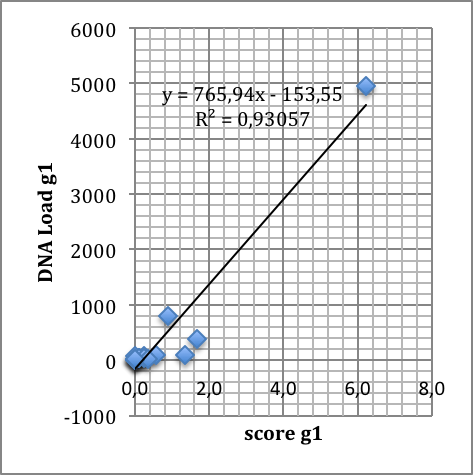


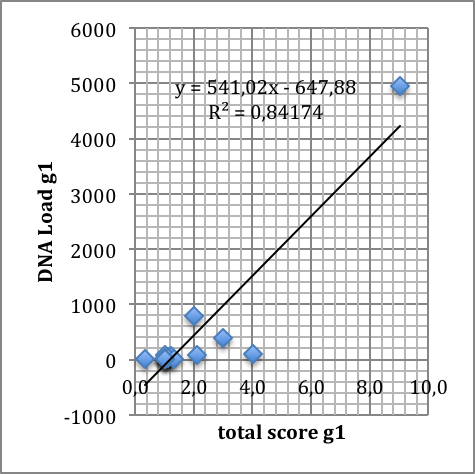

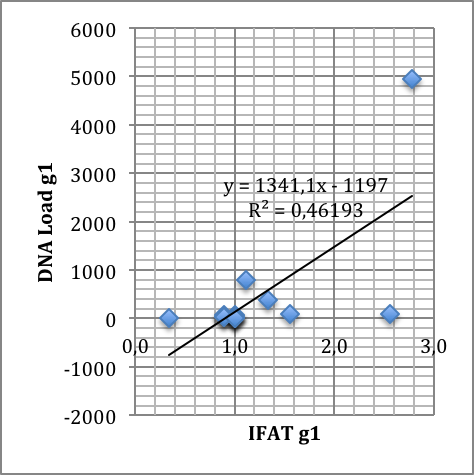


Figure S1: G1 dogs correlation study between clinical and laboratory parameters.


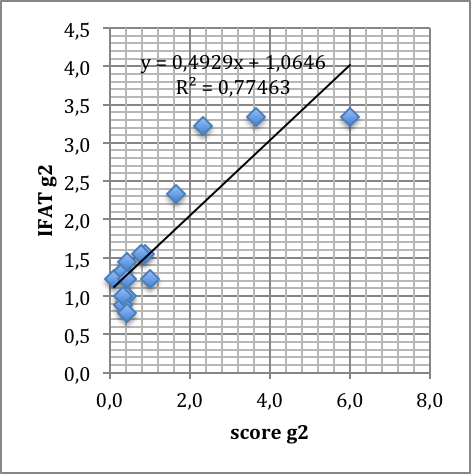

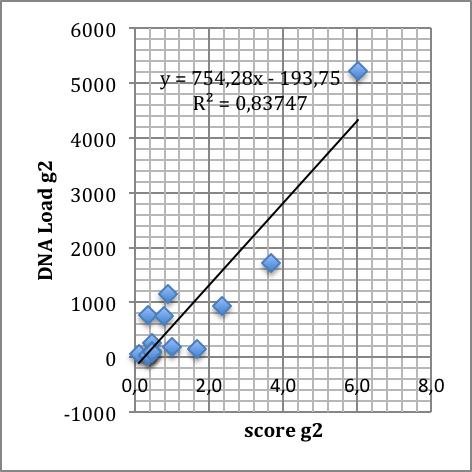


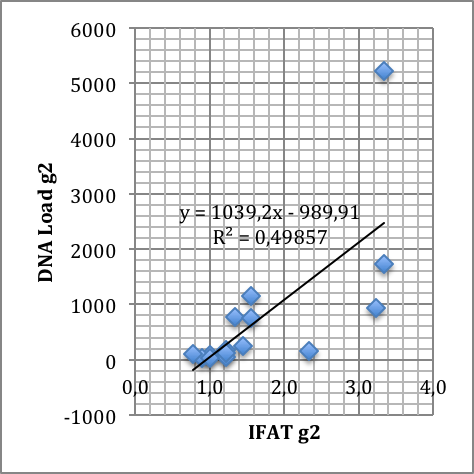


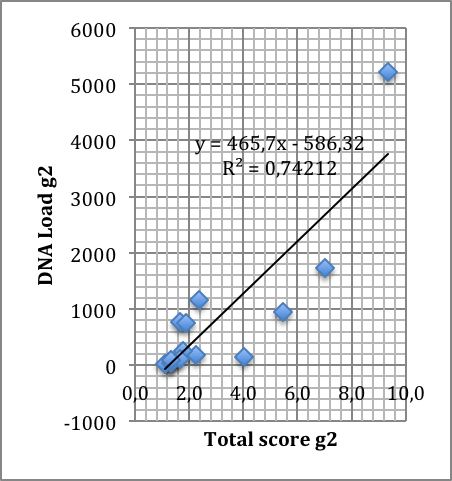


Figure S2: G2 dogs correlation study between clinical and laboratory parameters.
